# Association between health empowerment and medication literacy among older adults with coronary heart disease after PCI: the mediating role of self-efficacy for appropriate medication use

**DOI:** 10.3389/fpubh.2026.1877407

**Published:** 2026-10-14

**Authors:** Yuting Fan, Yuxin Li, Xipei Chen, Xiaoli Zhong, Ping Dai, Jijun Wu, Lin He

**Affiliations:** 1 Department of Nursing, Deyang People’s Hospital, Deyang, Sichuan, China; 2 School of Nursing, Chengdu University of Traditional Chinese Medicine, Chengdu, Sichuan, China; 3 Department of Cardiology, Deyang People’s Hospital, Deyang, Sichuan, China

**Keywords:** coronary heart disease, older adults, health empowerment, mediating effect, medication literacy, percutaneous coronary intervention, self-efficacy

## Abstract

**Background:**

With the acceleration of global population ageing and the increasing burden of chronic diseases, the number of older adults with coronary heart disease is steadily increasing. Medication literacy is an important foundation for safe medication use and is closely associated with medication management and secondary prevention among older adults with CHD after percutaneous coronary intervention. However, research on medication literacy in this population remains relatively limited, and factors associated with medication literacy have not been sufficiently investigated. Therefore, further research is needed to examine the factors associated with medication literacy among older adults with CHD after PCI, which may help inform future targeted intervention studies and the development of appropriate clinical interventions.

**Objective:**

To investigate the status of medication literacy among older adults with coronary heart disease after PCI, examine the association between health empowerment and medication literacy, and further assess the indirect association of self-efficacy for appropriate medication use in this relationship.

**Methods:**

This study adopted a cross-sectional survey design. Using convenience sampling, 483 older adults with coronary heart disease after PCI were recruited from two Grade A tertiary hospitals in Sichuan Province, China, from March to October 2025. On-site questionnaire surveys were conducted. The research instruments included the demographic characteristics, patient perceptions of empowerment scale, self-efficacy for appropriate medication use scale, and medication literacy scale for patients after PCI. Statistical analyses were performed using SPSS 26.0 and its PROCESS macro program.

**Results:**

Among the 483 older adults with coronary heart disease after PCI in this study, the score of health empowerment was 36.69 ± 6.19, the score of self-efficacy for appropriate medication use was 28.25 ± 4.73, and the score of medication literacy was 70.62 ± 17.82. Pearson correlation analysis showed that medication literacy was positively correlated with health empowerment (*r* = 0.810, *p* < 0.01) and self-efficacy for appropriate medication use (*r* = 0.821, *p* < 0.01), while health empowerment was positively correlated with self-efficacy for appropriate medication use (*r* = 0.811, *p* < 0.01). Mediation analysis showed a statistically significant indirect association between health empowerment and medication literacy through self-efficacy for appropriate medication use, with an indirect effect of 0.346. This indirect effect accounted for 47.92% of the total effect (95% CI: [0.287, 0.410]). In addition, the total effect was 0.722 (95% CI: [0.666, 0.777]), and the direct effect was 0.376 (95% CI: [0.299, 0.452]).

**Conclusion:**

Medication literacy among older adults with coronary heart disease after PCI was at a moderate level. Health empowerment, self-efficacy for appropriate medication use, and medication literacy were significantly and positively associated with one another, and a statistically significant indirect association between health empowerment and medication literacy through self-efficacy for appropriate medication use was observed. These findings suggest that healthcare professionals should consider health empowerment and self-efficacy for appropriate medication use when developing strategies to improve medication literacy among older adults after PCI, which may provide a reference for future intervention studies.

## 1 Introduction

In recent years, the pace of population aging in China has continued to accelerate. Relevant data shows that by 2025, China’s population aged 60 and older had reached 323 million, and is projected to exceed 500 million by 2050 (1). As aging intensifies, the burden of chronic diseases is growing increasingly severe. Among these, coronary heart disease, as one of the major chronic noncommunicable diseases threatening the health of older adults, has received widespread attention in recent years (2). According to reports, as of 2022, the prevalence of CHD among people aged 60 and older in China was as high as 27.8%, with incidence and mortality rates continuing to rise (3). This not only severely impacts patients’ health and well-being but also places a heavy burden on families and society. Percutaneous coronary intervention (PCI) is currently an effective clinical treatment for CHD, offering advantages such as “minimal invasiveness, rapid recovery, and significant therapeutic effects” (4). However, PCI can only address localized coronary artery stenosis and cannot fundamentally reverse the progression of atherosclerosis (5, 6). Patients still require long-term or even lifelong medication after the procedure to prevent adverse cardiovascular events, such as in-stent restenosis and thrombosis (7). Therefore, standardized and safe pharmacotherapy is crucial for disease management and long-term prognosis in patients who have undergone PCI for CHD.

Medication literacy is an important indicator for evaluating an individual’s ability to use medications safely and effectively (8). It refers to the capacity of individuals to acquire, understand, communicate, calculate, and process medication-related information, and thereby make scientific and rational medication decisions (9). Research indicates that high medication literacy enables patients to perform complex medication management tasks, reduce inappropriate medication use, and lower the risk of readmission (10). However, various factors—such as age-related cognitive decline, the burden of polypharmacy, and insufficient social support—contribute to low medication literacy among older adults (11, 12). This is reflected in a lack of medication knowledge, insufficient medication skills, limited ability to discern information, and poor adherence, all of which significantly increase the risk of adverse events like medication errors (13). For older adults who have undergone PCI for coronary heart disease, medication literacy is even more critical, as they require long-term, complex postoperative regimens (14). Therefore, thoroughly exploring the influencing factors and mechanisms underlying medication literacy in older adults who have undergone PCI is crucial for improving medication safety and reducing the risk of inappropriate medication use.

Health empowerment is closely associated with higher medication literacy (15). It refers to the process by which individuals fully mobilise their initiative to actively acquire health-related knowledge and skills to control disease, manage their lives, and promote health (16). Its benefits have been demonstrated in various healthcare contexts. Hsieh et al. (17) found that empowerment-based interventions could promote patients’ active participation in medication management and enhance their motivation for appropriate medication use, thereby supporting patients in actively acquiring and mastering medication-related knowledge. A study by Du et al. (18) also confirmed that higher levels of health empowerment can better stimulate patients’ initiative and inner potential, enhance their sense of responsibility, and encourage them to actively learn about medication-related knowledge. Based on this, we proposed hypothesis 1: Health empowerment is significantly associated with medication literacy among older adults with coronary heart disease after PCI.

Self-efficacy is a vital psychological resource that bridges cognition, motivation, and behavioral change, and it is another key factor influencing medication literacy (19). Self-efficacy for appropriate medication use, as a specific manifestation of self-efficacy in this context, focuses primarily on an individual’s confidence in their ability to take medication as prescribed (20). A firm belief and positive attitude form the foundation for acquiring knowledge and skills. A high sense of self-efficacy can motivate patients to learn about medication use and to actively follow treatment guidelines (21). A study by Wu et al. (22) also found that patients with higher levels of self-efficacy for appropriate medication use had greater confidence in their medication-taking behaviors and were more likely to actively seek and learn medication-related information. Furthermore, Social Cognitive Theory emphasizes that self-efficacy is an important psychological mechanism linking individual cognition with health behaviors (23). Based on this theory, health empowerment, reflected in active participation, perceived control, and self-management, may represent patients’ agency and active engagement in health management. When patients are more actively involved in managing their own health and have a stronger sense of control over their health, they may develop more positive perceptions of their health management capabilities. This positive individual experience may further strengthen their beliefs in their ability to manage medications appropriately, thereby promoting patients’ active acquisition, understanding, and application of medication-related information, enhancing medication literacy, and supporting appropriate medication use. Therefore, self-efficacy for appropriate medication use may play a potential mediating role in the association between health empowerment and medication literacy.

Currently, previous studies have separately reported associations among health empowerment, self-efficacy, and medication literacy (18, 22). However, no study has systematically examined the relationships among these three variables within the same research framework, particularly in the specific population of patients after PCI. Therefore, based on Social Cognitive Theory, we proposed the following hypotheses: (1) health empowerment is positively associated with medication literacy among older adults with coronary heart disease after PCI; and (2) self-efficacy for appropriate medication use may mediates the relationship between health empowerment and medication literacy among older adults with coronary heart disease after PCI. The hypothesized model is presented in Figure 1.

**Figure 1:**
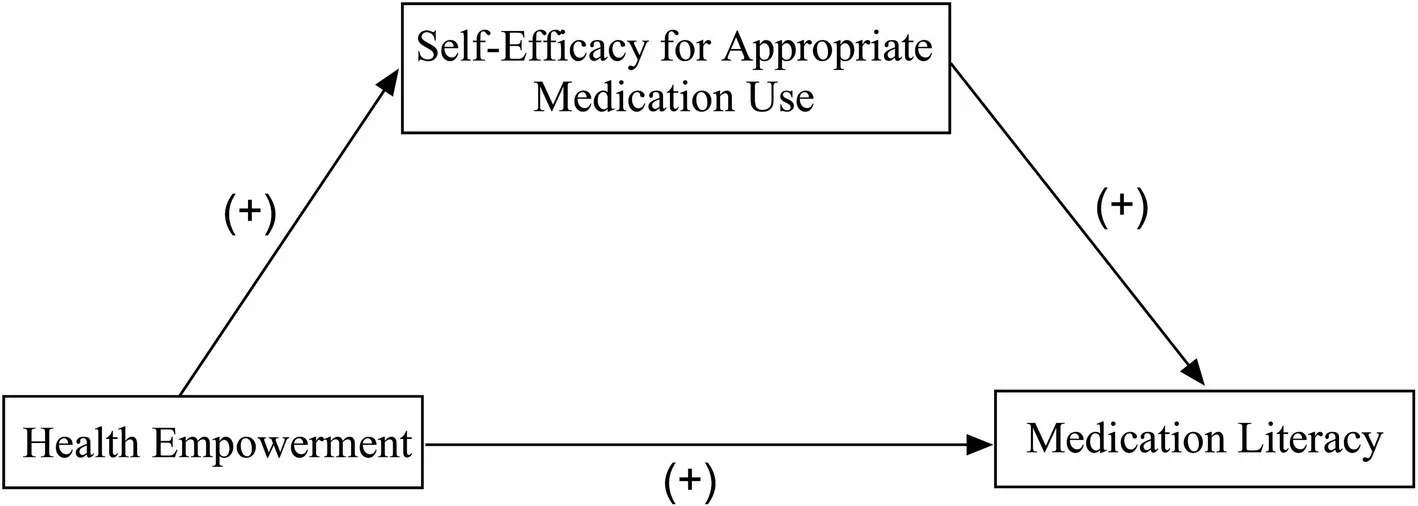
Diagram of model assumptions.

## 2 Methods

### 2.1 Study design

This study is a cross-sectional survey.

### 2.2 Research participants

This study used convenience sampling. From March to October 2025, participants who had undergone PCI for coronary heart disease were chosen. Participants came from the cardiovascular departments of two Grade A Level 3 hospitals in Chengdu and Deyang, Sichuan Province, China, to participate in an on-site questionnaire survey.

Inclusion Criteria: Participants who (1) met diagnostic and treatment criteria for coronary heart disease (24), had undergone PCI, and were attending outpatient follow-up; (2) were aged 60 or older; (3) had stable conditions, clear consciousness, and could understand and communicate; (4) provided informed consent and participated voluntarily.

Exclusion criteria: (1) Severe comorbidities involving other organ systems, such as vital organ dysfunction (e.g., liver, brain, kidneys), severe infections, or malignant tumours; (2) Severe hearing, visual, or cognitive impairment, or psychiatric disorders; (3) Current participation in other drug-related studies.

The required sample size was calculated using G*Power 3.1 software (25). In accordance with Cohen’s guidelines (26), the statistical power was set at 0.95, the significance level (α) at 0.05, and the effect size (f^2^) at 0.15 (moderate effect size). A total of 22 predictors were included in the calculation, comprising 16 demographic variables, four dimensions of patient-perceived empowerment, and two dimensions of self-efficacy for appropriate medication use. The calculated minimum required sample size was 230 participants. Considering a potential 20% rate of invalid questionnaires, the minimum target sample size was adjusted to 288 participants. Ultimately, 483 patients were included in the analysis, yielding an effective response rate of 98.57%. Figure 2 illustrates the process of selecting research subjects.

**Figure 2:**
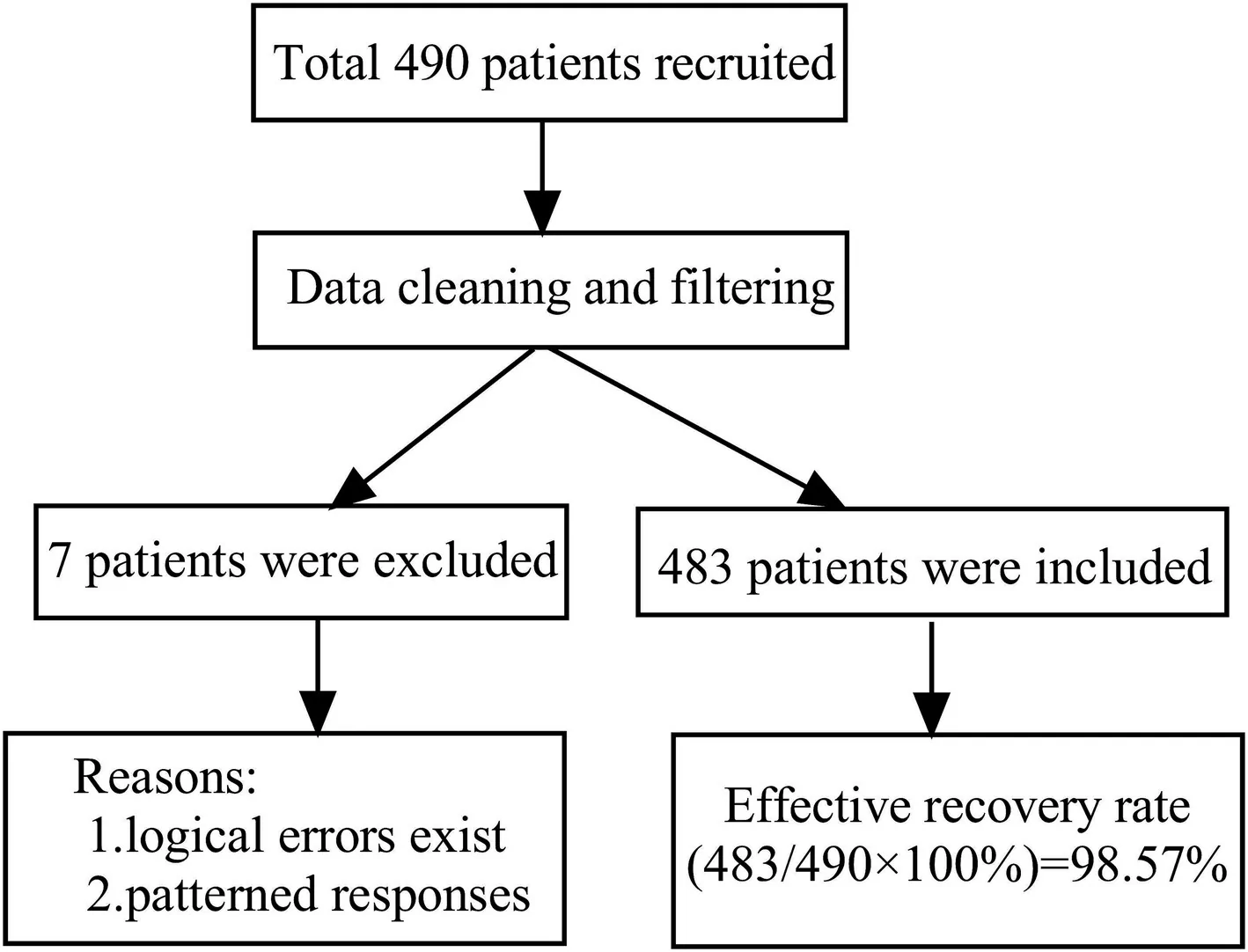
Participant flowchart.

### 2.3 Measurement tools

#### 2.3.1 The demographic characteristics

This questionnaire was designed by the research team in accordance with the objectives of this study, based on an extensive review of relevant literature and fully tailored to the specific needs of this research. Specifically, it includes: gender, age, level of education, marital status, place of residence, occupational status, type of medical insurance, average monthly household income, whether a family members supervise medication intake, whether received education on safe medication use, duration of illness, number of stents implanted, number of comorbid chronic conditions, duration of medication use, number of medications taken, and daily medication frequency.

#### 2.3.2 Patient perceptions of empowerment scale

The Chinese version of the Patient Perceptions of Empowerment Scale, adapted by Yeh et al. (27) in 2014, was used for assessment. The scale comprises four dimensions (information, decision-making, personalisation, and self-management) and 11 items. A 5-point Likert scale was used, with scores ranging from 1 to 5, from “strongly disagree” to “strongly agree.” The total score ranges from 11 to 55, with higher scores indicating greater health empowerment. In this study, the Cronbach’s alpha coefficient for this scale was 0.744.

#### 2.3.3 Self-efficacy scale for appropriate medication use

The assessment was conducted using the Self-Efficacy Scale for Appropriate Medication Use Scale, developed by Risser et al. (28) in 2002 and adapted into Chinese by Dong et al. (29) in 2015. The scale covers two dimensions—medication use under uncertainty and under difficult circumstances—and has 13 items, each rated on a 3-point Likert scale from “not at all confident” to “very confident,” assigning scores of 1 to 3, respectively. The total score ranges from 13 to 39, with higher scores indicating stronger self-efficacy in adhering to rational, standardised medication regimens. In this study, the Cronbach’s alpha coefficient for this scale was 0.813.

#### 2.3.4 Medication literacy scale for patients after percutaneous coronary intervention

The Medication Literacy Scale for Patients after Percutaneous Coronary Intervention, developed by Zhang et al. (30) in 2024, was used for assessment. This scale comprises five dimensions (medication beliefs, functional medication literacy, interactive medication literacy, evaluative medication literacy, and behavioral medication literacy) and 25 items in total. In particular, medication beliefs reflect patients’ knowledge of medicines and their understanding of the benefits and risks associated with their appropriate use; functional medication literacy reflects the ability to understand and apply medication-related knowledge; interactive medication literacy refers to the ability to actively seek medication-related information and communicate with others about medication use; critical medication literacy reflects the ability to analyse and evaluate medication-related information; and behavioral medication literacy reflects the ability to follow medical advice and use medicines appropriately in practice. A 5-point Likert scale was used, ranging from “not at all aware” to “fully aware,” scored from 1 to 5. The total score ranges from 25 to 125, with the following scoring categories: 25–60 points indicate low medication literacy, 61–81 points indicate moderate medication literacy, and 82–125 points indicate high medication literacy. In this study, the Cronbach’s alpha coefficient for this scale was measured at 0.937.

### 2.4 Data collection

In this study, questionnaires were collected on-site through face-to-face interviews. Approval was obtained from the hospital’s Nursing Department and relevant department heads prior to the survey. The research team, uniformly trained by the principal investigator in interview techniques and data entry standards, aimed to ensure data accuracy and consistency. During the survey, participants were rigorously screened using the inclusion and exclusion criteria, and questionnaires were distributed only after informed consent was obtained. Using a standard set of instructions, trained researchers explained the survey’s purpose, the methods for completing it, and key points to respondents. For participants who were unable to complete the questionnaire independently because of reading or writing difficulties, trained researchers read the items verbatim and provided standardized, non-leading explanations when necessary, without suggesting or influencing responses. Respondents could withdraw from the study at any time. Completed questionnaires were collected on-site and immediately checked for completeness. If any issues were identified, they were promptly verified and resolved with the respondent.

### 2.5 Data analysis

This study utilised SPSS 26.0 statistical software and the Process 3.5.2 plugin for data analysis. First, the normality of continuous variables was assessed based on skewness and kurtosis. The absolute values of skewness were less than 3 and those of kurtosis were less than 10, indicating that the data were approximately normally distributed. Therefore, continuous variables were expressed as means ± standard deviations, while categorical variables were described using frequencies and percentages. Independent-samples *t*-tests or one-way ANOVA were used to compare group differences. Pearson correlation analysis was used to examine the correlations among continuous variables. Variance inflation factors (VIFs) were used to assess potential multicollinearity among the variables. Multivariate hierarchical regression analysis was conducted to identify factors associated with medication literacy, and the potential mediating role of self-efficacy for appropriate medication use in the association between health empowerment and medication literacy was preliminarily examined. For the mediation analysis, Model 4 in the Process 3.5.2 plugin was used, with health empowerment as the independent variable, medication literacy as the dependent variable, and self-efficacy for appropriate medication use as the mediator. Covariates were initially identified based on the results of the univariate analyses, and their potential clinical relevance to medication literacy among older adults with coronary heart disease after PCI was further considered in the subsequent analyses. The 95% confidence interval was estimated using the Bootstrap method with 5,000 bootstrap samples, and a two-tailed test was conducted at α = 0.05.

### 2.6 Ethical considerations

Throughout this study, we strictly adhered to the principles of informed consent, confidentiality of participant information, and non-maleficence. This study complies with the Declaration of Helsinki and received approval from the Ethics Committees of Chengdu University Affiliated Hospital and Deyang People’s Hospital prior to its formal commencement. (Ethics Approval Numbers: PJ 2024-118-01 and 2025-04-042-K01).

## 3 Results

### 3.1 Common method bias test

Since the data in this study were derived from self-report scales, the participants’ subjective evaluations may introduce common-method bias. To enhance the rigour of this study, Harman’s one-factor test was used to conduct an unrotated exploratory factor analysis of the items from the scales involved. The results showed that nine factors had a total eigenvalue greater than 1. The first factor explained 33.54% of the cumulative variance, which was below the standard cutoff of 40%, and this did not indicate substantial common-method variance in this study.

### 3.2 Relationship between general characteristics of study participants and medication literacy

Among the 483 older adults with coronary heart disease who underwent PCI included in this study, the majority were male (64.0%), the largest age group was 60 to <70 years (44.1%), and the majority had an educational level of elementary school or below (60.0%). See Table 1 for the remaining general characteristics.

**Table 1:** Demographic characteristics of older adults with coronary heart disease after PCI and their relationship to medication literacy.

Project		*N*(%)	Medication Literacy		
			Mean ± SD	*t/F*	*p*
Gender	Male	309 (64.0%)	70.12 ± 18.00	−0.818	0.413
	Female	174 (36.0%)	71.51 ± 17.52		
Age (years)	60 ~ <70	213 (44.1%)	70.03 ± 18.36	0.216	0.806
	70 ~ <80	191 (39.5%)	71.02 ± 18.07		
	≥80	79 (16.4%)	71.27 ± 15.76		
Level of education	Primary school and below	290 (60.0%)	69.61 ± 17.99	1.201	0.302
	Junior high school	116 (24.0%)	72.43 ± 17.68		
	High school/technical secondary school and above	77 (15.9%)	71.69 ± 17.34		
Marital status	Married	421 (87.2%)	70.83 ± 18.03	0.675	0.500
	Single/Divorced/Widowed	62 (12.8%)	69.19 ± 16.37		
Place of residence	Rural	255 (52.8%)	70.02 ± 17.73	−0.789	0.430
	Towns	228 (47.2%)	71.30 ± 17.93		
Occupational status	Retirement	420 (87.0%)	70.74 ± 17.61	0.372	0.710
	Currently employed	63 (13.0%)	69.84 ± 19.31		
Type of medical insurance	Urban Resident Medical Insurance	166 (34.4%)	72.30 ± 17.17	4.056	0.018
	Urban and Rural Residents’ Medical Insurance	270 (55.9%)	70.75 ± 17.86		
	Self-funded/Other	47 (9.7%)	63.98 ± 18.70		
Average monthly household income (yuan)	<3,000	228 (47.2%)	66.11 ± 17.45	16.133	<0.001
	3,000 ~ 5,000	215 (44.5%)	73.86 ± 17.31		
	>5,000	40 (8.3%)	78.93 ± 16.12		
Whether a family member supervises medication intake	Yes	218 (45.1%)	72.78 ± 17.55	2.432	0.015
	No	265 (54.9%)	68.84 ± 17.88		
Whether received education on safe medication use	Yes	263 (54.5%)	73.16 ± 18.09	3.468	0.001
	No	220 (45.5%)	67.58 ± 17.04		
Number of comorbid chronic diseases	1	187 (38.7%)	76.82 ± 18.13	26.432	<0.001
	2	172 (35.6%)	69.61 ± 16.59		
	≥3	124 (25.7%)	62.68 ± 15.53		
Duration of illness (years)	<5	161 (33.3%)	65.67 ± 17.02	24.479	<0.001
	5 ~ <10	192 (39.8%)	68.93 ± 17.83		
	≥10	130 (26.9%)	79.25 ± 15.70		
Number of stents implanted	1	225 (46.6%)	72.20 ± 17.97	2.565	0.078
	2 ~ 3	223 (46.2%)	69.83 ± 17.81		
	≥4	35 (7.2%)	65.51 ± 16.03		
Duration of medication use (years)	<1	117 (24.2%)	73.74 ± 18.24	2.446	0.063
	1 ~ <5	215 (44.5%)	70.13 ± 18.42		
	5 ~ <10	73 (15.1%)	66.73 ± 17.20		
	≥10	78 (16.1%)	70.95 ± 15.40		
Number of medications taken	<3	157 (32.5%)	77.69 ± 15.75	30.826	<0.001
	3 ~ 5	156 (32.3%)	71.62 ± 18.28		
	≥6	170 (35.2%)	63.18 ± 16.34		
Daily medication frequency	1	119 (24.6%)	72.95 ± 17.75	5.296	0.005
	2	208 (43.1%)	72.11 ± 17.89		
	≥3	156 (32.3%)	66.87 ± 17.29		

### 3.3 Univariate analysis of relation between general characteristics and medication literacy

Univariate analysis shows that medication literacy among older adults with coronary heart disease after PCI was significantly associated with type of medical insurance, average monthly household income, family supervision of medication, education on safe medication use, number of comorbid chronic conditions, duration of illness, number of medications taken, and daily medication frequency (all *p* < 0.05). See Table 1 for details.

### 3.4 Descriptive and correlational analysis of health empowerment, self-efficacy for appropriate medication use, and medication literacy

Among 483 older adults with coronary heart disease who underwent PCI, the mean health empowerment score was 36.69 ± 6.19, the mean self-efficacy for appropriate medication use score was 28.25 ± 4.73, and the mean medication literacy score was 70.62 ± 17.82. Pearson correlation analysis showed positive correlations among all three variables: health empowerment and medication literacy (*r* = 0.810, *p* < 0.01), health empowerment and self-efficacy for appropriate medication use (*r* = 0.811, *p* < 0.01), and self-efficacy for appropriate medication use and medication literacy (*r* = 0.821, *p* < 0.01) (see Table 2).

**Table 2:** Descriptive and correlational analysis of health empowerment, self-efficacy for appropriate medication use, and medication literacy.

Variables	Mean±SD	1(*r*)	2(*r*)	3(*r*)
1. Health empowerment	36.69 ± 6.19	1		
2. Self-efficacy for appropriate medication use	28.25 ± 4.73	0.811^**^	1	
3. Medication literacy	70.62 ± 17.82	0.810^**^	0.821^**^	1

^**^p < 0.01; *r* is the correlation coefficient (Pearson correlation).

### 3.5 Hierarchical multiple regression analysis

First, variables that were statistically significant in the univariate analysis of medication literacy were entered into the model as control variables. In the second step, after controlling for the relevant variables (type of medical insurance, average monthly household income, whether a family members supervise medication intake, whether received education on safe medication use, number of comorbid chronic diseases, duration of illness, number of medications taken, and daily medication frequency), health empowerment was further entered into the model. The results showed that health empowerment significantly improved the explanatory power of the model, with the proportion of variance explained increasing by 39.2% compared with the first step (*β* = 0.721, *SE* = 0.085, *p* < 0.001). In the third step, self-efficacy for appropriate medication use was further entered into the model based on the variables included in the previous steps, resulting in an additional 6.7% increase in the proportion of variance explained compared with the second step (*β* = 0.450, *SE* = 0.147, *p* < 0.001). Additionally, the results of the multicollinearity diagnosis indicated that the tolerance values for each model ranged from 0.324 to 0.968 (all > 0.1), and the VIF values ranged from 1.033 to 3.090(all < 5), indicating that there is no multicollinearity among the variables. See Table 3 for details.

**Table 3:** Multivariate hierarchical regression analysis of medication literacy among older adults with coronary heart disease after PCI.

Control variable	Model 1				Model 2				Model 3			
	*SE*	*β*	*t*	*p*	*SE*	*β*	*t*	*p*	*SE*	*β*	*t*	*p*
Type of medical insurance	1.147	−0.048	−1.206	0.228	0.762	0.006	0.242	0.809	0.675	0.016	0.681	0.496
Average monthly household income	1.147	0.129	3.147	0.002	0.773	0.006	0.228	0.820	0.683	0.003	0.140	0.889
Whether a family member supervises medication intake	1.395	−0.087	−2.239	0.026	0.926	−0.055	−2.124	0.034	0.819	−0.049	−2.130	0.034
Whether received education on safe medication use	1.408	−0.068	−1.720	0.086	0.939	0.004	0.145	0.884	0.832	0.021	1.408	−0.068
Number of comorbid chronic diseases	0.893	−0.247	−6.226	<0.001	0.608	−0.095	−3.507	<0.001	0.542	−0.060	−2.498	0.013
Duration of illness (years)	0.906	0.221	5.604	<0.001	0.618	0.066	2.443	0.015	0.546	0.059	2.484	0.013
Number of medications taken	0.854	−0.267	−6.764	<0.001	0.577	−0.139	−5.202	<0.001	0.511	−0.126	−5.325	<0.001
Daily medication frequency	0.925	−0.100	−2.575	0.010	0.614	−0.062	−2.389	0.017	0.544	−0.049	−2.133	0.033
Health empowerment					0.085	0.721	24.633	<0.001	0.114	0.377	9.550	<0.001
Self-efficacy for appropriate medication use									0.147	0.450	11.515	<0.001
F	25.656				119.369				150.585			
R^2^	0.302				0.694				0.761			
adjusted *R^2^*	0.290				0.688				0.756			
*p*	<0.001				<0.001				<0.001			

### 3.6 Mediation analysis among health empowerment, self-efficacy for appropriate medication use, and medication literacy

Standardization was performed for all variables. Mediation analysis was conducted using Model 4 in Process v3.5. Health empowerment was the independent variable, medication literacy the dependent variable, and self-efficacy for appropriate medication use the mediating variable. Control variables included whether a family member supervises medication intake, number of comorbid chronic conditions, duration of illness, number of medications taken, and daily medication frequency.

Health empowerment was positively associated with self-efficacy for appropriate medication use (*a* = 0.774, *SE* = 0.029, *p* < 0.001). Health empowerment was positively associated with medication literacy (*c′* = 0.376, *SE* = 0.039, *p* < 0.001), and self-efficacy for appropriate medication use was positively associated with medication literacy (*b* = 0.447, *SE* = 0.039, *p* < 0.001). The Bootstrap analysis showed that the direct effect of health empowerment on medication literacy was 0.376 (95% *CI* [0.299, 0.452]), while the total effect was 0.722 (95% *CI* [0.666, 0.777]). The indirect effect of health empowerment on medication literacy through self-efficacy for appropriate medication use was 0.346 (95% *CI* [0.287, 0.410]). As the 95% *CI*s for all three effects did not include zero, these findings indicated a statistically significant indirect association between health empowerment and medication literacy through self-efficacy for appropriate medication use. The indirect effect accounted for 47.92% of the total effect. See Table 4; Figure 3.

**Table 4:** Results of mediation analysis.

Effects	Effect size	*SE*	95% *CI*
Total effect			
Health empowerment→Medication literacy	0.722	0.028	(0.666, 0.777)
Direct effect			
Health empowerment→Medication literacy	0.376	0.039	(0.299, 0.452)
Indirect effect			
Health empowerment→Self-efficacy for appropriate medication use→Medication literacy	0.346	0.031	(0.287, 0.410)

**Figure 3:**
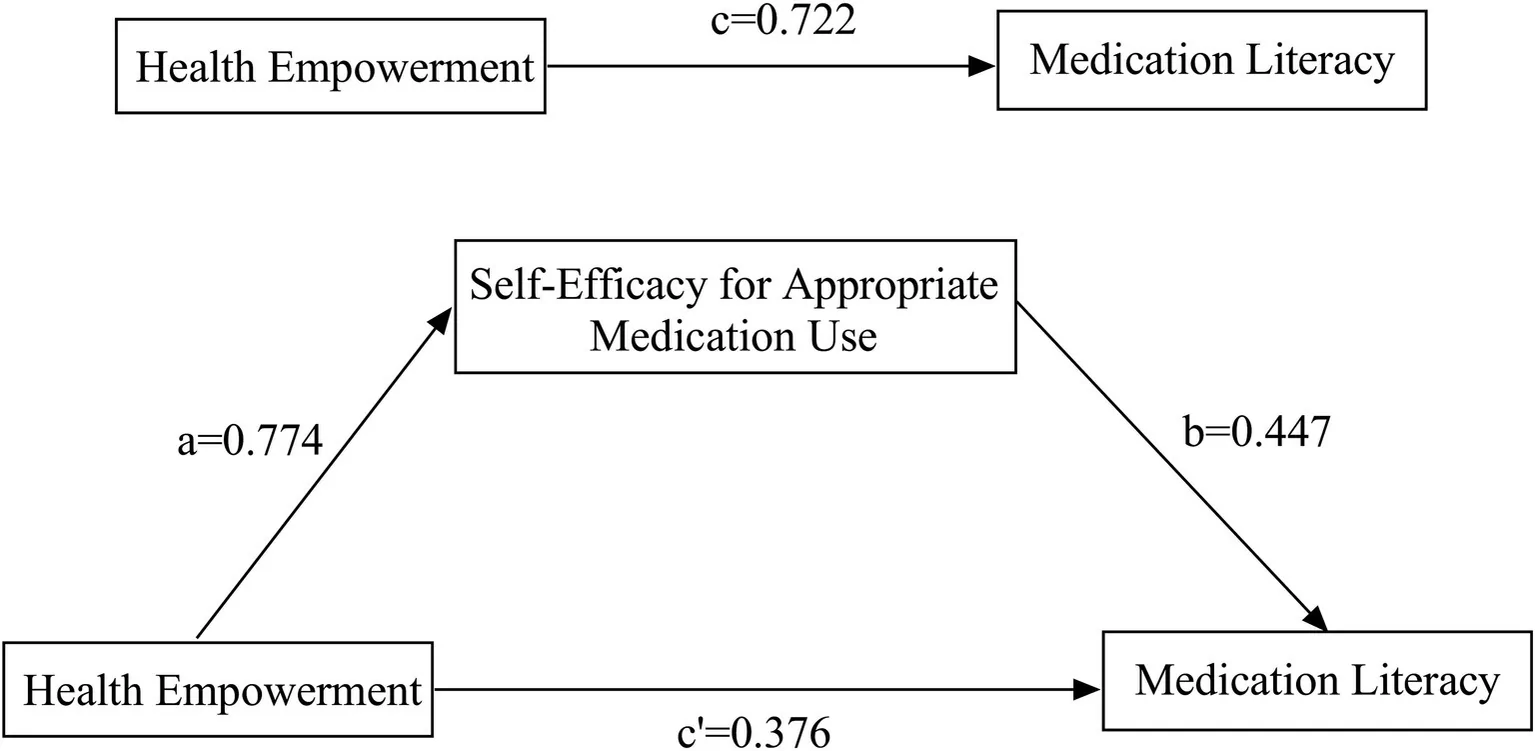
Schematic diagram of the mediational analysis of self-efficacy for appropriate medication use between health empowerment and medication literacy.

## 4 Discussion

### 4.1 Current status of medication literacy among older adults with coronary heart disease after PCI

The results of this study showed that the medication literacy score among older adults with coronary heart disease after PCI was 70.62 ± 17.82. According to the classification criteria of the scale, the medication literacy of older adults after PCI was at a moderate level. Further analysis showed that family supervision of medication intake, duration of illness, number of comorbid chronic conditions, number of medications taken, and daily medication frequency were associated with medication literacy among older adults with coronary heart disease after PCI. Possible explanations for these associations are as follows: (1) Family members may help patients maintain regular medication-taking behaviors through daily supervision and reminders, reducing inappropriate medication-taking behaviors such as self-reduction of doses or medication discontinuation (31). In addition, family members’ care may provide emotional support and positive encouragement, facilitate patients’ participation in medication management and health-related decision-making, and may be associated with higher medication literacy (32). (2) A greater number of comorbid chronic conditions generally means that patients need to manage multiple diseases simultaneously, with potentially more complex medication goals, treatment regimens, and precautions (33). This may increase the difficulty of understanding and processing medication-related information among older adults with coronary heart disease after PCI (34, 35). (3) Older adults with coronary heart disease after PCI who had a longer disease duration had relatively higher levels of medication literacy, which may be related to the medication-related experience accumulated during long-term treatment (36). (4) Older adults with coronary heart disease after PCI who took a greater number of medications and had a higher daily medication frequency had relatively lower levels of medication literacy. A possible explanation is that increases in the number and frequency of medications often require patients to invest more time, effort, and financial resources (37). Such a greater medication management burden may be accompanied by psychological stress, with patients showing relatively less initiative in learning medication-related information and managing their medications (38). These circumstances may be associated with lower levels of medication literacy.

### 4.2 Correlation analysis of health empowerment, self-efficacy for appropriate medication use, and medication literacy

This study found a positive correlation between health empowerment and medication literacy (*r* = 0.810, *p* < 0.01), indicating that higher levels of health empowerment were associated with higher levels of medication literacy among older adults following PCI. This finding is consistent with that reported by Hu et al. (39). This positive association may be related to the active participation, autonomous decision-making, and self-management characteristics inherent in health empowerment. Specifically, on the one hand, patients with higher levels of health empowerment may pay greater attention to their health status and medication regimens, participate more actively in medication-related decision-making and daily medication management, and seek and learn relevant information to enhance their understanding of prescribed medications (40). On the other hand, health empowerment emphasises patients’ active participation in their own health management, which may facilitate their expression of medication-related needs and concerns and encourage more active communication with healthcare professionals. Through such interactions, patients may obtain more targeted and comprehensible medication information, which may further facilitate their understanding and appropriate application of medication-related information (41). Furthermore, previous studies have also suggested that effective patient–provider communication can help patients clarify the purpose of medication and improve their understanding of medication-related information, thereby supporting appropriate medication use (42).

This study found that health empowerment is significantly positively correlated between health empowerment and self-efficacy for appropriate medication use (*r* = 0.810, *p* < 0.01), indicating that higher levels of health empowerment were associated with stronger self-efficacy for appropriate medication use among older adults after PCI. This finding is consistent with those reported in previous studies (43). Health empowerment emphasises a patient-centred approach and prioritises the individual needs of older adults; by providing support for disease understanding and creating opportunities for active participation, it can enhance their self-esteem and self-confidence (44). Another study also indicates that higher levels of health empowerment can strengthen patients’ active engagement, encourage them to express their own needs, and stimulate intrinsic motivation, thereby fostering a sense of agency in the medication management process (16). Establishing this sense of agency helps patients develop positive beliefs about medication adherence, which, in turn, enhances their confidence in managing medication-related issues and increases their self-efficacy (45).

This study found that self-efficacy for appropriate medication use was significantly positively correlated with medication literacy (*r* = 0.821, *p* < 0.01), indicating that higher levels of self-efficacy for appropriate medication use were associated with higher levels of medication literacy among older adults after PCI. This finding is consistent with the results reported by Wang et al. (46). According to self-efficacy theory, self-efficacy refers to an individual’s confidence in their ability to perform a specific behavior in a given situation, which directly influences their behavioral choices, level of effort, and persistence (47). Higher self-efficacy for appropriate medication use may give patients greater confidence in managing medication-related issues, making them more likely to proactively seek solutions and devote time and effort to learning about medicines, obtaining and understanding medication information, and addressing medication-related problems during actual medication use (48). Furthermore, Research by Bakan et al. (49) also indicates that individuals with higher self-efficacy were more likely to adopt proactive problem-solving strategies, actively seek medication-related information, and identify potential risks. These findings may help explain the observed positive association between self-efficacy for appropriate medication use and medication literacy in the present study.

However, it is worth noting that the overall correlations among the three variables were relatively strong in the present study (*r* = 0.810–0.821). These correlation coefficients appeared to be higher than those reported in previous studies, which may be related to differences in the study population, clinical context, and measurement instruments.

### 4.3 The mediating role of self-efficacy for appropriate medication use between health empowerment and medication literacy

This study found a significant indirect association between health empowerment and medication literacy through self-efficacy for appropriate medication use among older adults with coronary heart disease after PCI. The indirect association accounted for 47.92% of the total effect. Supporting hypothesis 2. Specifically, health empowerment centers on facilitating patients’ transition from “passively receiving treatment” to “actively participating in health management.” When patients are encouraged to participate in treatment-related decision-making, they may develop a stronger sense of autonomy and control over their health, which may enhance their confidence in managing their disease and medication treatment and, in turn, be associated with higher self-efficacy for appropriate medication use (50). Self-efficacy reflects an individual’s positive belief in their ability to successfully perform a specific behavior and may facilitate patients’ active seeking, understanding, and application of medication-related information (51). In addition, previous studies have indicated that, compared with patients with lower self-efficacy, those with higher self-efficacy tend to have stronger medication-related beliefs, more positive attitudes toward medication use, and a greater willingness to acquire medication-related knowledge (52). According to Social Cognitive Theory (23), self-efficacy is a core factor linking individual characteristics with health-related outcomes. From this perspective, on the one hand, better health empowerment may be closely associated with improvements in medication-related knowledge, medication-taking skills, and awareness of appropriate medication use among patients with chronic diseases. On the other hand, patients with higher levels of health empowerment may have a stronger sense of control and greater confidence in coping with challenges related to disease management and medication treatment, which may encourage them to actively seek medication-related information and critically evaluate such information, thereby being associated with higher levels of medication literacy.

This suggests that healthcare professionals should place greater emphasis on the concept of patient empowerment in clinical practice, actively encourage older adults with coronary heart disease after PCI to recognize themselves as the primary person responsible for their own health, strengthen their self-efficacy for appropriate medication use, and improve their medication literacy.

### 4.4 Implications for clinical practice

The results of this study indicate that health empowerment, self-efficacy for appropriate medication use, and medication literacy were associated with one another among older adults with coronary heart disease after PCI; furthermore, a statistically significant indirect association between health empowerment and medication literacy was observed through self-efficacy for appropriate medication use. Based on these findings, these variables may be considered when developing and evaluating future strategies to improve medication literacy among older adults with coronary heart disease after PCI.

At the individual level, patients could be encouraged to take an active role in enhancing their understanding of their own disease and medications, including their therapeutic effects, proper use, and precautions, while developing awareness of appropriate medication use and participating actively in medication management. During daily medication use, patients could be encouraged to pay attention to their medication-taking practices, record changes in their medication regimen, communicate with healthcare professionals, and independently acquire medication-related knowledge to strengthen their sense of agency in medication management. These approaches may be considered as potential components of future interventions aimed at supporting medication literacy.

At the healthcare professional level, incorporating the concept of patient empowerment into medication health education and strengthening medication information support may represent areas worthy of further exploration in future medication health education. Specifically, healthcare professionals could consider patients’ individual circumstances and encourage their active participation in medication management, such as through jointly developing medication plans. For example, medication-related knowledge after PCI could be explained in clear and easy-to-understand language to help patients better access and comprehend medication information. For older adults, clearer and more concise approaches to presenting medication information could be explored, such as appropriately adjusting font size and information layout, to facilitate access to and reading of medication-related content (53). In addition, for patients who lack confidence in medication use, future interventions could explore the incorporation of communication and feedback opportunities to allow patients to express concerns and share their experiences with medication use.

## 5 Limitations

This study has several limitations that should be considered. First, the cross-sectional design primarily reflects the status of the variables and their associations at a specific point in time and therefore cannot determine the temporal sequence or causal relationships among the variables. Future longitudinal or interventional studies are warranted to further examine the dynamic changes in these variables and their potential relationships over time. Second, convenience sampling was used, and the participants were mainly recruited from healthcare institutions in Chengdu and Deyang, Sichuan Province. The relatively concentrated sample sources may have limited the representativeness and generalizability of the findings to some extent. Therefore, caution should be exercised when generalizing these findings to older adults after PCI in other regions, healthcare settings, or countries. Future multicenter and cross-regional studies with broader and more diverse samples are needed to further validate and extend the findings of this study. Third, all variables in this study were assessed using self-reported questionnaires and collected at the same time point, which may have introduced recall bias and social desirability bias. Although the Harman single-factor test was used to provide a preliminary assessment of common method bias, this approach has certain methodological limitations; therefore, the findings should be interpreted with caution. Future studies may incorporate objective measures, multiple data sources, or assessments at multiple time points to further reduce the potential impact of common method bias.

## 6 Conclusion

In summary, this study investigated the current status of medication literacy among older adults with coronary heart disease after PCI and examined the associations among health empowerment, self-efficacy for appropriate medication use, and medication literacy. The findings demonstrated significant associations among health empowerment, self-efficacy for appropriate medication use, and medication literacy, together with a statistically significant indirect association between health empowerment and medication literacy through self-efficacy for appropriate medication use. Future longitudinal or interventional studies are needed to further examine the temporal and causal relationships among health empowerment, self-efficacy for appropriate medication use, and medication literacy, as well as to dynamically track changes in these variables over time. Based on the indirect association pathway identified in this study, future research may further develop and validate approaches targeting patients’ health empowerment and self-efficacy for appropriate medication use, which may provide a reference for developing comprehensive strategies centered on patients’ active engagement in health management and aimed at improving medication literacy.

## Data Availability

The raw data supporting the conclusions of this article will be made available by the authors, without undue reservation.
