# Correction: Association between health empowerment and medication literacy among older adults with coronary heart disease after PCI: the mediating role of self-efficacy for appropriate medication use

**DOI:** 10.3389/fpubh.2026.2030812

**Published:** 2026-10-23

**Authors:** Yuting Fan, Yuxin Li, Xipei Chen, Xiaoli Zhong, Ping Dai, Jijun Wu, Lin He

**Affiliations:** 1 Department of Nursing, Deyang People's Hospital, Deyang, Sichuan, China; 2 School of Nursing, Chengdu University of Traditional Chinese Medicine, Chengdu, Sichuan, China; 3 Department of Cardiology, Deyang People's Hospital, Deyang, Sichuan, China

**Keywords:** coronary heart disease, older adults, health empowerment, mediating effect, medication literacy, percutaneous coronary intervention, self-efficacy

In the published article, there was an error regarding the affiliations for Xipei Chen and Ping Dai.

Xipei Chen's correct affiliation should be “School of Nursing, Chengdu University of Traditional Chinese Medicine, Chengdu, Sichuan, China”, instead of “Department of Nursing, Deyang People's Hospital, Deyang, Sichuan, China”.

Ping Dai's correct affiliation should be “Department of Cardiology, Deyang People's Hospital, Deyang, Sichuan, China”, instead of “Department of Nursing, Deyang People's Hospital, Deyang, Sichuan, China”.

The original version of this article has been updated.

